# Innate Functions of Dendritic Cell Subsets in Cardiac Allograft Tolerance

**DOI:** 10.3389/fimmu.2020.00869

**Published:** 2020-05-05

**Authors:** Samantha Schroth, Kristofor Glinton, Xunrong Luo, Edward B. Thorp

**Affiliations:** ^1^Department of Pathology and Feinberg Cardiovascular and Renal Research Institute, Feinberg School of Medicine, Northwestern University, Chicago, IL, United States; ^2^Department of Medicine, School of Medicine, Duke University, Durham, NC, United States; ^3^Department of Pediatrics, Feinberg School of Medicine, Northwestern University, Chicago, IL, United States

**Keywords:** dendritic cell, transplant, tolerance, innate, cardiac

## Abstract

Survival rates after heart transplant have significantly improved over the last decade. Nevertheless, long-term allograft viability after 10 years remains poor and the sequelae of transplant-associated immunosuppression increases morbidity. Although several studies have implicated roles for lymphocyte-mediated rejection, less is understood with respect to non-major histocompatibility, and innate immune reactivity, which influence graft viability. As immature and mature dendritic cells (DCs) engage in both Major Histocompatibility Complex (MHC)-dependent and MHC-independent immune responses, these cells are at the crossroads of therapeutic strategies that seek to achieve both allograft tolerance and suppression of innate immunity to the allograft. Here we review emerging roles of DC subsets and their molecular protagonists during allograft tolerance and allograft rejection, with a focus on cardiac transplant. New insight into emerging DC subsets in transplant will inform novel strategies for operational tolerance and amelioration of cardiac vasculopathy.

## Clinical Relevance

The volume of heart transplants performed worldwide has continued to rise as surgical transplantation remains a standard of care for patients with advanced heart failure (1). According to a recent report, 3,273 heart transplants were performed in the United States in 2017 in addition to a continued increase in new listings for transplantation (2). While cutting-edge surgical tools and advances in immunosuppression have improved acute posttransplant mortality, significant morbidity is still experienced by heart transplant recipients.

Chronic allograft vasculopathy (CAV), an accelerated version of atherosclerosis characterized by diffuse thickening of arterial walls, remains a noteworthy cause of long-term graft attrition with 29.3% of transplant recipients experiencing CAV 5 years post-transplant and an astounding 47.4% of patients within 10 years (3). While the specific pathogenesis of CAV has yet to be fully elucidated, a significant factor recognized to contribute to CAV is a maladaptive immune response (4, 5). Thus, the need for improved strategies to suppress chronic immune reactivity to the allograft remains.

Numerous studies have attributed chronic rejection to lymphoid and antibody-mediated mechanisms where graft reactive antibody constitute a persistent inflammatory state. Experimental and clinical data indicate that graft-reactive antibody triggers inflammatory signaling by graft endothelium and interstitial cells, thereby activating donor-reactive, and non-specific innate and adaptive immune mechanisms (6). Such persistent inflammatory insults subvert natural wound healing pathways and may lead to a state of non-resolving inflammation often associated with chronic graft interstitial fibrogenesis and vascular injury. This inflammatory response is difficult to resolve and therefore a key obstacle in preventing progression of chronic graft injury.

Relative to studies on T lymphocytes, less is appreciated regarding the role of innate immune cells, including monocytes, dendritic cells (DCs), and macrophages, following solid organ transplantation and their involvement in this chronic inflammatory state. While these cells are classically thought of as first responders, emerging evidence encourages us to reevaluate this “surface level” thinking and consider a deeper investigation as events occurring early after transplant have the potential to contribute to or even perpetuate long term damage. While much could be said about each of the innate immune cell types, our discussion will focus specifically on the role of DCs in heart transplantation.

## DC Subsets

DCs are canonically recognized as professional antigen presenting cells (APCs) that serve as a key linking cell between the non-specific innate immune response and the memory producing, antigen specific adaptive immune response. As such, DCs are a remarkably heterogenous population of cells, existing in a variety of subtypes that differ in surface phenotype, function, and location in the body (7). Attempts to accurately classify DCs into their appropriate subtypes has been fraught with challenges as cell surface markers commonly used in classification schemes are often not unique to a particular cell subtype and vary based upon activation state of a cell or location in the body. A nomenclature for DC subsets based primarily on ontogeny and secondarily by location, function, and phenotype was proposed by Guilliams et al. (8) which we will apply in an attempt to maintain clarity in this discussion.

**Figure 1:**
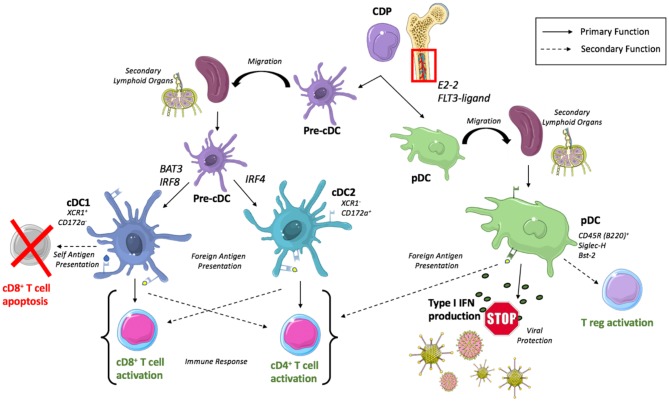
Development of DC subsets and conically associated functions. cDCs arise from a common dendritic cell precursor (CDP) originating in the bone marrow. CDPs mature into pre-cDCs and then migrate to secondary lymphoid organs where they differentiate into cDC1s or cDC2s dependent upon transcription factors, BAT3/IRF8 and IRF4, respectively. cDC1s are recognized to predominantly activate CD8^+^ T cells but secondarily can induce CD8^+^ T cell apoptosis with the presentation of self-antigen while cDC2s predominantly activate CD4^+^ T cells. pDCs complete their maturation within the bone marrow before migrating to secondary lymphoid organs where they participate in type I IFN production for viral protection and participate (to a lesser extent) in antigen presentation.

DCs develop from a common progenitor cell in the bone marrow into classical DCs (cDCs) with potent antigen presenting abilities, or plasmacytoid DCs (pDCs) implicated in the production of type I interferons (IFNs) and subsequent innate immunity against viral infection (7) (Figure 1). Recent evidence has emerged suggesting very early separation of these lineages during development, where pDC precursor cells differentiate from a lymphoid progenitor cell that is independent of the myeloid cDC lineage (9). However, it is important to note a fair amount of disagreement exists as to the identity of a true DC progenitor that exclusively gives rise to the DC lineage and ensuing subsets. Nevertheless, our appreciation of DC subset heterogeneity and lineage is undergoing continuous evolution driven by current studies that leverage transcriptomic and single cell sequencing, coupled with genetic lineage tracing.

Additionally, there is some controversy concerning a final category of monocyte-derived DCs (moDC) which are routinely generated *in vitro* (10) but are defined uniquely by researchers as either macrophage-like or DC-like based upon expression of CD11c; further scenarios complicated by inflammation alters this phenotypic profile (8). However, moDC have remained of interest to researchers due to their use in DC vaccination immunotherapy for cancer treatment (11). Below we focus our discussion on cDC and pDC subsets.

### Classical Dendritic Cells

Following migration of a committed precursor cell (pre-cDC) from the bone marrow to peripheral lymphoid and non-lymphoid tissues (12), cDCs will complete their development into cDC1 and cDC2 subsets dependent upon a unique set of transcription factors where BATF3 and IRF8 have been recognized as crucial for regulation of cDC1 development (13, 14) and IRF4 for cDC2s (15, 16). These subsets can be differentiated by surface markers across multiple tissues as XCR1^+^ Cadm1^+^ CD172a^−^ cDC1s and XCR1^−^ Cadm1^−^ CD172a^+^ cDC2s (17), or with additional tissue specific markers such as splenic CD8α^+^ cDC1 and CD4^+^ cDC2 or lung CD103^+^ cDC1 and CD11b^+^ cDC2.

The predominate function of cDCs is recognized to be antigen presentation, where XCR1^+^ CD172^−^ cDC1s present to and subsequently stimulate a CD8^+^ T cell response (18) while XCR1^−^ CD172^+^ cDC2s are more adept at stimulating CD4^+^ helper T cells and humoral immunity (19). Importantly, DC subsets exhibit remarkable plasticity dependent upon their microenvironment (20), allowing for XCR1^−^ CD172^+^ cDC2s and pDCs to retain the ability to cross-present antigens to CD8^+^ T cells when appropriately stimulated (21, 22).

### Plasmacytoid Dendritic Cells

The development of pDCs requires the transcription factor E2-2 (23) and is regulated by cytokine FLT3-ligand in both mice and humans (24, 25). Unlike cDCs, development of pDCs is completed in the bone marrow prior to their migration to secondary lymphoid organs and peripheral tissues. The complex biology of pDC has been reviewed extensively by others (26), however a brief overview of their unique phenotype and functionality is warranted.

Identification of pDCs requires the use of multiple surface markers in order to accurately delineate a pure pDC population. Murine pDCs are known to express CD11c (though at lower levels than cDCs), CD45R (B220), Sca-1, Siglec-H, Bst2, and CCR9 in addition to markers that are thought to be related to maturation state such as Ly6C, CD4, and CD8 (27).

Functionally, activated pDCs are able to perform the canonically associated antigen presenting role of a DC, however they do so much less efficiently than cDCs (28, 29). pDCs exhibit a lower expression of MHC class II and costimulatory molecules compared to their cDC counterparts, but mature pDCs are still able to generate an effective, and immunogenic T cell response (30). This response has been revealed to be variable, polarizing to direct Th1 or Th2 differentiation dependent upon factors including antigen dose, stimulation type, and cell maturation state (31).

With these somewhat “weak” antigen presenting capabilities and ability to prime T cells, pDCs are more recognized for their role in production of type I Interferon in response to viral stimulation (32). This subset specific high level production of type I interferon is known to activate NK cells yielding induction of cytotoxicity and IFN-γ production (33), helping to orchestrate the TLR9 mediated control of viral infection (34).

Beyond this predominant function of cytokine production, it has been suggested that given appropriate stimuli, pDC are able to induce the development of CD4^+^CD25^+^ regulatory T cells (Tregs) as demonstrated following co-culture of CD4^+^CD25^−^ naïve T cells with pDCs enriched from human peripheral blood mononuclear cells (PBMCs) (35). Relatedly, pDCs have been shown to activate resting CD4^+^CD25^+^Foxp3^+^ Tregs *in vivo* isolated from murine tumor draining lymph nodes in an indoleamine 2,3-dioxygenase (IDO)^+^ pDC dependent manner (36).

## Innate Response of DCs in Cardiac Transplant

As we begin to assess the innate response of the aforementioned DC subsets in cardiac transplant, it is important to consider the environment these cells currently or will soon occupy. Organ transplantation induces rapid activation of the innate immune system as damaged vascular and parenchymal tissue from organ procurement, organ storage, and engraftment yield numerous inflammatory stimuli derived from dead or dying graft cells. These released damage-associated molecular patterns (DAMPs) are then recognized by toll-like receptors (TLRs), a type of pattern-recognition receptor (PRR), which initiate a signaling cascade that results in production of multiple cytokines and cellular responses to further enhance this inflammatory milieu (37).

The onslaught of immune cell infiltration has long been assumed to be damaging to the graft with cell-specific depletion studies further confirming. Depletion of macrophages in a mouse model of heart transplantation revealed markedly reduced development of CAV lesions (4) and a related attenuation of experimental transplant vasculopathy has been documented in association with reduced numbers of graft-associated macrophage and dendritic cells (38). Complimentarily, the presence of intravascular macrophages within the first year after human heart transplant was found to be predictive of donor specific antibody and potential development of antibody mediated rejection (39). However, a subset of macrophages have also been shown to play a role in angiogenesis and may aid in microvascular repair of the injured graft (40). Thus, we must ask, how is it that DCs contribute to the innate response following cardiac transplantation?

The innate immune response is composed of physical, cellular, and chemical components that function as a first line defense to protect the body from invading pathogens and/or foreign antigens. As such, the innate response can be thought to consist of elements that respond directly to this invader and elements responsible for signaling to members of the adaptive response, allowing for formation of immunological memory (41). DCs have revealed that they participate in both of these elements, in many ways serving as a cellular director who orchestrates the recruitment of necessary cell populations dependent upon the pathology occurring.

Immature DCs specialize in the capture and processing of antigens, after which they mature and lose much of their endocytic capacity while upregulating the expression of MHCII and other costimulatory molecules such as CD40, CD80, and CD86 (42). Engagement with either MHCII or CD40 on DCs has long been known to result in their production of IL-12 (43) which plays an important role in the activation of NK cells and their subsequent production of IFN-γ (44) known to regulate Th1 cell development (45). This interplay between DCs and NK cells within the innate immune system has garnered attention of recent as increasing numbers of molecular and cell-to-cell interactions between these two cells are uncovered. A cellular conversation occurring reciprocally between DCs and NK cells has been found to result in a diversity of outcomes including NK cell activation and proliferation as well as either DC maturation or elimination [reviewed by Degli-Esposti and Smyth (46)]. The importance of NK cell activation in transplant has been argued by data showing adoptive transfer of NK cells 1 day prior to heart transplant in T/B/NK-deficient mice resulted in the development of CAV, which was not seen in mice who remained NK cell deficient (47). While it was not an objective of this study to assess the involvement of DCs in this NK-cell required pathology, it is important to consider the importance and presence of upstream cellular signals that may allow for the end result. Additionally, when we consider the conversation that occurs between NK cells and DCs in the context of transplantation, we must add an additional layer as both recipient and donor “passenger” immune cells of both cell types could be involved. However, It has been demonstrated that recipient NK cells will quickly eliminate donor allogeneic DCs found within the transplanted organ (48), indicating it is likely recipient DCs that play the predominant role in the innate response.

Continuing with the concept of DCs as cellular director of the innate response, elegant experiments from the lab of Florent Ginhoux have identified skin cDC1s (distinct from epidermis associated antigen presenting Langerhans cells) as producers of the cytokine VEGF-α, recognized to be important in the recruitment of neutrophils (49). Depletion of this DC subset via diphtheria toxin injection yielded a significant decrease in neutrophils to the cutaneous injury site which could be recovered following cDC1 adoptive transfer. Interestingly, neutrophils isolated from cDC1 depleted mice revealed a downregulation of genes associated with priming, mobility, and neutrophil recruitment compared to their cDC1 sufficient counterparts. Additionally, neutrophils from a cDC1 deficient environment exhibited decreased functional capacity and survival. The recency of this study have not allowed for evaluation of this finding in other pathologies, such as the setting of transplant. However, given that a low lymphocyte to neutrophil ratio was shown to be a potential biomarker to predict acute rejection after heart transplant (50), understanding the way in which cDC1s participate in neutrophil recruitment in this setting may be of immense value.

Having discussed the direct responses of DCs to foreign antigen, the second category of elements in the innate response to consider is methods of signaling to members of the adaptive immune system to initiate a primary immune response. While other innate immune cells are recognized for their phagocytic properties (namely macrophages), the responsibility of uptake and subsequent presentation of foreign bodies to lymphocytes to trigger an adaptive response fall on DCs. The efficiency by which different DC subsets present antigen to T cells has already been mentioned, however it is important to recognize the complexity which underlies antigen presentation and allorecognition in the setting of donor and recipient immune cells.

A series of potential allorecognition pathways amongst DC and T cells exist including direct, indirect, and semi-direct allorecognition. Direct allorecognition refers to the recognition of MHC-peptide complexes on donor APCs directly by recipient T cells (51) which likely only play a role during acute graft rejection, as donor-derived APCs will eventually die or be destroyed, prohibiting them from participating in chronic forms of rejection (52). Meanwhile, indirect allorecognition occurs when recipient DCs process graft derived peptides and present these molecules on self-MHC to lymphocytes (53) which has been shown to play a role in both acute and chronic rejection in multiple models (54–57). Finally, the semi-direct pathway involves transfer of intact donor MHC molecules to recipient DCs in a process also referred to as “MHC cross-dressing” by mechanisms still being defined such as cellular “nibbling,” also known as trogocytosis, or DC secreted exosomes (58–61). These related but disparate pathways by which recipient or donor DCs uptake or receive graft antigen to utilize in signaling to the adaptive immune system speak of the versatility of this cell within the innate response, A final topic within the innate response of DCs following transplantation worthy of mention is the recognition of allogenic self vs. non-self. Classically, the innate immune system relies upon recognition of conserved microbial molecular patterns also known as pathogen-associated molecular patterns (PAMPs) by PRRs for identification of non-self to subsequently trigger an immune response (62). But what is the innate role of self vs. non-self recognition within the setting of sterile inflammation where PAMPs do not play a role, as occurs in transplant? It has been found that mice devoid of T, B, and natural killer cells are still able to mount an immune response via innate recognition of allogenic non-self (63). This recognition of non-self is shown to be the result of allelic polymorphisms in donor SIRPα membrane protein on donor tissue binding to CD47 (or IAP for integrin-associated protein) on recipient infiltrating DCs (64). Continuing to assess and improve our understanding of the magnitude and effect of this innate non-self recognition response, mediated by DCs, in the setting of solid organ transplant may have important future clinical implications for organ allocation.

## DCs in Transplant Tolerance

A worthy goal in the realm of organ transplantation is the induction of operational tolerance in which there is long-term survival of an allograft without need for immunosuppressive therapies (65). This is an attractive objective, as current pharmacological agents commonly used for maintenance immunosuppression in heart transplant are recognized to result in severe side effects including (but not limited to) nephrotoxicity, dyslipidemia, pancytopenia, and pericardial and pleural effusions (66). Thus, addressing the ability of DCs to induce a tolerogenic state following solid organ transplantation is of great value. Here we will seek to evaluate how DCs and their subsets may play a role in this induction of operational tolerance.

It has also been noted that the relative composition of DC subsets found in the peripheral blood following heart transplantation is mutable, dependent upon factors such as choice of pharmacologic immunosuppressant and length of time since transplant (67). In a study of human heart transplant recipients, an association between lower levels of pDCs and increased rejection grades was observed (68). However, it is important to note this study evaluated two groups of patients treated with different immunosuppressive therapies, tacrolimus (TAC), and cyclosporine A (CsA). The authors report patients treated with TAC have significantly higher values of pDCs than CsA treated patients in addition to decreased rejection. It is very possible there are multiple pharmacologic mechanisms contributing to the rejection phenotype observed, but the DC subset specific differences between these populations is intriguing.

A number of studies have begun to further probe this interesting observation by adoptively transferring pDCs into rodent models of heart or lung transplant and observing graft outcome [reviewed in Rogers et al. (69)]. Remarkably, a consistent story of prolonged graft survival with pDC adoptive transfer begins to emerge. Relatedly, depletion of donor pDCs from bone marrow grafts resulted in accelerated graft-vs.-host disease (GVHD) mortality (70). A similar result was observed in murine heart transplant in which treatment with tolerizing protocol followed by pDC depleting antibody prevented tolerance induction (71). This study further describes the localization of pDCs to high endothelial venules in the lymph nodes with a related distribution of Treg cells. Additional experiments by the authors reveal pDCs to promote Treg development, a cell recognized to play a role in both induction and maintenance of tolerance (reviewed in Tang and Vincenti (72)].

More recently, the cDC1 subset has also begun to reveal its own unique role in the context of tolerance. In a mouse model of peripheral tolerance assessing the renal lymph node, a site where self and foreign antigen are continuously filtered, cDC1 cells were shown to induce apoptosis of CD8^+^ cytotoxic T cells through programmed death 1 ligand (PD-L1) signaling (73). While this study evaluates cDC1 driven apoptosis as a mechanism to prevent auto-immunity, the potential implications in transplant are readily apparent as a means to deplete graft reactive T cells, although this, to the authors' knowledge, has yet to be formally assessed.

cDC1s have also been found to play a vital role in central tolerance by presentation of cell-surface antigens from apoptotic medullary thymic epithelial cells yielding development of a diverse repertoire of regulatory T cell receptors (74). Interestingly, this CD36 dependent antigen transfer to CD8α^+^ DCs (equivalent to cDC1s) was shown to be required for thymic allo-tolerance in a murine model of GVHD following BMT. Implicating this pathway further, a blinded analysis of peripheral blood of patients following BMT revealed a correlation between decreased CD36 expression and CD141^+^ DCs (the human equivalent of CD8α^+^ cDC1s) with increased frequency of GVHD development, despite no significant differences in prevalence of other cell types, demographic, or clinical characteristics. While the thymic environment and processes associated with central tolerance are admittedly removed from tolerance induction following solid organ transplant, evaluating the mechanisms by which tolerance in various settings is successfully achieved in parallel with the involved cellular players may help to facilitate the generation of new tolerogenic therapies.

## DCs in Tolerance Inducing Therapies

A variety of protocols have been developed and refined to commandeer and direct the interactions of immune cells in a manner that would promote a tolerogenic environment following organ transplantation leading to graft acceptance. Of special interest to the authors is the use of donor cells, with or without additional combinatorial therapy, as a means to harness the body's ability to clear naturally occurring apoptotic cells via phagocytosis without damaging healthy neighboring cells or initiating an inflammatory milieu (75). We will discuss the currently known role of DCs in this process, but a broader discussion of the mechanisms that underly the use of apoptotic cell-based therapies in the promotion of tolerance can be found in the review by Morelli and Larregina (76).

Treatment of donor splenocytes with the chemical crosslinker 1-ethyl-3-(3-dimethylaminopropyl) carbodiimide (ECDI-SP) induces apoptosis, allowing for the processing and presentation of donor antigens in a non-immunogenic manner (77). Donor ECDI-SP is intravenously infused into the recipient 7 days before and 1 day after transplantation, resulting in indefinite survival of full MHC-mismatch allogenic pancreatic islet grafts (78) and prolonged survival of heart allografts in the absence of immunosuppression. Interestingly, when ECDI-SP treatment was combined with a short course of rapamycin (from day −1 to day +8), long term cardiac graft survival (>150 days) was achieved in all recipients (79). The differential success of this treatment strategy in two unique solid organ transplants could point toward an organ specific immune response, variations in mechanism for tolerance induction reliant upon organ resident or infiltrating immune cells, or other such hypotheses that must be addressed. Still, this tolerizing treatment strategy exhibits potential, revealing both safety and tolerability in a phase I trial of patients with MS (80) and in a phase I/IIa trial as prophylaxis for GVHD (81).

The role of DCs in this tolerizing protocol has been revealed to be essential, as only depletion of CD11c^+^ DCs via administration of diphtheria toxin to CD11c^+^ DTR mice was able to inhibit islet allograft survival following ECDI-SP treatment (82). In the same study, internalization of ECDI-SP was seen to occur with varying proportions across the various DC subsets, however all of these populations were simultaneously depleted in the CD11c^+^ DTR mouse model when defining the necessity of DCs. Additional research, such as the use of subset specific depletion models, are required to delineate if specific roles and/or differing levels of importance exist within the respective DC subsets in the induction and maintenance of ECDI-SP induced tolerance.

The manner in which DCs are impacted by ECDI-SP treatment deserves consideration as this knowledge may help to elucidate other powerful molecular targets for tolerance induction. Kheradmand et al. (82) reported an upregulation of negative costimulatory molecules, PD-L2, and PD-L1, with no increase in positive costimulatory molecules on CD11c^+^ DCs of mice treated with ECDI-SP, a balance which was essential for tolerance. The PD-1 pathway and its associated ligands are recognized to be involved in the T cell response (83) where engagement of PD-1 on activated T cells by its known ligands inhibits T cell proliferation (84, 85). Again, it has yet to be determined if the upregulation of these molecules (or others not investigated in this study) following ECDI-SP treatment are differentially attributed to specific DC subsets. This type of result would prompt preferential targeting of the identified subset in order to yield an enhanced response while minimizing off-target effects.

Other techniques to exploit DC interactions have been explored in which donor antigens, usually in the form of splenocytes, are delivered to recipients in conjunction with costimulation blockade (86, 87). Classically, naïve T cells require two signals for their activation and subsequent proliferation: (1) recognition of APC presented antigen by T cell receptor (2) costimulatory signal (88). Thus, the administration of antigen in combination with an antibody that blocks the required costimulatory signal, such as anti-CD154(CD40L), allows for an altered functional DC phenotype similar to that established in the setting of peripheral tolerance (89). This technique has been shown to yield robust tolerance in the setting of murine cardiac allograft transplantation, in which only a triple intervention strategy (depletion of Tregs, PD-L1 antagonism, low dose T cell transfer) was able to break established tolerance (90).

Some concern has been raised of clinical viability of the CD40-CD40L blockade strategy following the discovery of CD40L on endothelial cells and stimulated platelets (91) and a high incidence of thromboembolic events in primates after receiving monoclonal antibody against CD40L (92). However, a newly generated CD154 blocking antibody utilizing a mutated IgG1 construct lacking Fc activity was able to prolong kidney graft survival in a non-human primate without evidence of thromboembolic complications, helping to demonstrate that obstacles for clinical utility of this strategy can be overcome (93).

When CD40 (expressed on DCs) binds to CD40L (expressed on activated CD4^+^ T cells), a complex pathway of downstream signaling is initiated that alters DC phenotype and functionality in order to promote an effective T cell response. This includes increasing MHC and costimulatory molecule expression, increasing production of inflammatory cytokines (94), and encouraging DC longevity (95). Although costimulation blockade with CD154 occurs at the level of the T cell, it is important to consider how interrupting this DC to T cell interaction effects DCs due to the absence of the CD40:CD40L signal. Following CD40/CD154 blockade, (96) demonstrated a significant reduction in inflammatory cytokines secreted by DCs and delayed expansion and differentiation of host reactive T cells. However, somewhat surprisingly DCs were shown to express similar levels of positive costimulatory molecules (CD80, CD86) as untreated controls.

**Figure 2:**
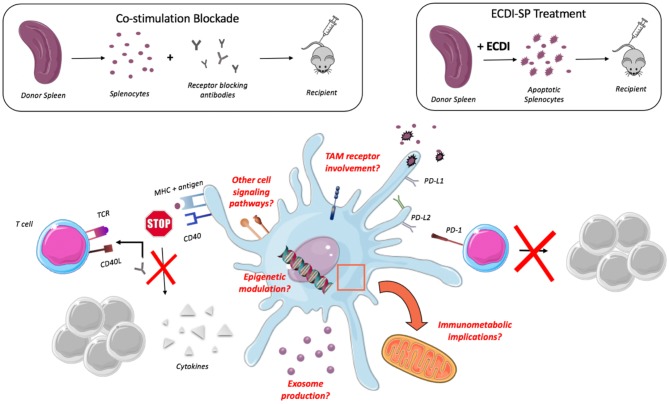
Impact of tolerogenic therapy on DCs. Administration of donor antigen with CD40L blocking antibodies results in decreased production of cytokines by DCs while administration of ECDI-induced apoptotic cells results in upregulation of negative costimulatory molecules (PD-L1, PD-L2) yielding an inhibition of T cell proliferation. Questions remain as to the role of TAM receptors, epigenetic modulation, exosome production, and Immunometabolic implications.

While both ECDI-SP and CD40-CD154 costimulation blockade strategies exhibit promise for inducing tolerance in transplant recipients, it's important to note such tolerance appears to be achieved through dissimilar mechanisms (upregulation of inhibitory molecules vs. reduced cytokine secretion) as far as DCs are concerned. Continuing to further delineate the cellular and molecular mechanisms by which tolerance is induced and maintained may allow for use of such therapies in combination and improved pharmacological targeting (Figure 2).

## DC Immunometabolism and Transplantation

Over the past 5 years there has been a developing interest in the relationship between cellular metabolism and its influence on cell function. Recent studies have shown alterations to metabolic pathways, including glycolysis, the Krebs cycle, and fatty acid metabolism, are able to profoundly influence the function of macrophages and DCs in notably specific ways (97). While metabolic pathways are unmistakably complex, the advent of increasingly sophisticated, and sensitive molecular tools have allowed us to begin to unravel this intricate network of potential therapeutic targets.

The bioenergetic requirements of DCs are highly dependent upon their activation or lack thereof, and yet viewing the concept of metabolism purely from an “energy providing” point of view is a vast oversimplification. Exciting work has revealed metabolites themselves, such as NAD^+^ and succinate, are able to provide signals to immune cells that regulate their function (98). For example, it is accepted that toll-like receptor (TLR) agonism is crucial for DC activation from its quiescent state. More interestingly, TLR agonism on DCs was shown to result in a metabolic transition from oxidative phosphorylation (inactive) to aerobic glycolysis (active) which could be inhibited by adenosine monophosphate activated protein kinase (AMPK) (99). This direct inhibition of DC activation by the hand of cellular metabolism should encourage us to postulate how immunometabolism contributes to a tolerogenic or rejecting DC phenotype in the setting of solid organ transplant.

With the help of deep mRNA sequencing and molecular pathway analysis software, it has been shown that tolerogenic DCs from human peripheral blood (induced by modulation with 1,25-dihydroxyvitamin D2 and dexamethasone) do indeed differentially express genes associated with metabolic pathways. Pathways of oxidative phosphorylation, lipid, and sugar metabolism were shown to be two-fold higher in these tolerogenic DCs compared to mature inflammatory DCs (100). Such findings have been seen by others while also observing that tolerogenic DCs express higher levels of proteins involved in mitochondrial fatty acid oxidation (FAO). Interestingly, blocking FAO blunted some of the tolerogenic function of these DCs as measured by increased levels of activated T cells following said blockade (101). It is unknown if these metabolic adaptations function consistently across the identified DC subsets, but this rapid cellular modification to alterations in the metabolic milieu resulting in observable change in the tolerogenic capacity of DCs certainly encourage further investigation.

## Therapeutic Implications and Future Research

This basic understanding of the powerful role DCs play at the intersection of innate and adaptive immunity in combination with strategies already showing therapeutic potential are an encouragement to the pursuit of achieving operational tolerance. Additional strategies and future research directions should consider complementary or supplemental interrogation of regulatory DC receptors that have yet to be evaluated.

The TAM family of receptor tyrosine kinases—TYRO3, Axl, and Mer—are expressed among cells of the immune system including macrophages, resting and activated DCs, and natural killer cells. These receptors are recognized to play essential roles in innate immunity including inhibition of the inflammatory response, phagocytosis of apoptotic cells, and maturation of natural killer cells (102). However, in the case of Axl and Mer, these roles have been identified as diverging with Axl expression increasing following inflammatory stimuli and Mer expressed on resting macrophages and enhanced following tolerogenic stimuli, such as in culture with immunosuppressive dexamethasone (103). Zagorska et al. (103) identified *in vitro* bone marrow-derived DCs as having greater levels of inflammation associated Axl in comparison to Mer, a finding further supported by similar levels of expression on CD11c^+^ DCs isolated from murine spleen. Characterization of these receptors within specific DC subsets has not performed and use of gene and cell specific knockout models or receptor specific antibodies could aid in elucidating targetable pathways amongst the TAM receptors for use in transplant.

Currently identified ligands of the TAM receptors include growth-arrest specific six protein (GAS6) and Protein S (104). These proteins serve as so-called “linker molecules” to the TAM receptor as they are simultaneously bound to phosphatidylserine present on apoptotic cell membranes. Interestingly, in a murine model of autoimmune thyroiditis, the prevalence of thyroiditis, and inflammatory infiltrate was shown to be significantly decreased in mice that received recombinant Gas6 (105). These mice also showed distinct differences in the distribution of T cell subsets following treatment with Gas6, however though APCs are required for T cell activation, the impact of Gas6 administration on APCs was not evaluated.

A potential strategy toward harnessing the selective activation of the aforementioned receptors may be through the use of nanobiologics that selectively target DCs, such as dendritic cell-targeted polymersomes (106). This idea of selectively targeting innate immune cells has already begun to be investigated with promising results. Braza et al. (107) utilized a short term high-density lipoprotein nanobiologic to encapsulate the mTOR inhibitor, rapamycin in order to preferentially target myeloid cells, and inhibit trained immunity. Significantly higher uptake of these particles was seen in macrophages compared to other cell types (DCs, neutrophils) with a related decrease in TNFα and IL-6 protein expression by flow sorted macrophages from mice receiving this non-biologic treatment. Perhaps most exciting is treatment with this myeloid targeting nanobiologic yielded significantly increased heart allograft survival, even when compared to oral and intravenously administered rapamycin. Thus, it appears combining cell-specific targeting and nanobiologic treatment could serve as a powerful tool in the promotion of tolerance.

## Concluding Remarks

In this review, we have sought to highlight what is currently known as it relates to DCs in the setting of cardiac transplantation. We highlight this unique cell as a prominent director of the innate immune response with the ability to activate and recruit additional cell types, such as NK cells and neutrophils, and participate in allogenic recognition and signaling to the adaptive system. We describe DC subset classification and what has been found regarding subset specific roles in transplantation tolerance where both pDCs and cDC1s could serve as important cellular mediators of tolerance, although this has yet to be fully elucidated. We assess the impact of tolerization therapies, such as delivery of apoptotic cells or costimulation blockade, acting on DCs in related albeit dissimilar mechanisms through upregulation of inhibitory molecules vs. reduced cytokine secretion, respectively. Finally, we address areas in which research has only just begun including the implications of immunometabolic modulation and the interrogation of regulatory DC receptors such as the TAM family with nanobiologics.

As our ability to probe deeper into specific immune cell populations continues to advance, we find ourselves at a unique time in immunology to be able to ask questions and address pathways and cellular functions in ways like never before. There has been a drastic increase in understanding of the innate immune system over the past few years, yet gaps in knowledge certainly remain within the realm of transplantation and DCs. Future directions of research require careful consideration of subset specific receptors and subsequent responses in order to better delineate DC roles in tolerance and maximize potential therapeutic targets. We must also deepen our awareness of the mechanisms by which DCs mediate both the adaptive and innate response in transplant, evaluating DC signaling utilized for cellular recruitment, innate sensing of self vs. non-self, and receptors necessary for tolerogenic DC programming. With a growing and evolving knowledge of this complex cell and the innate immune system, true transplant tolerance, considered to be the “holy grail” of transplant research, may become within reach.

## Author Contributions

All authors listed have made a substantial, direct and intellectual contribution to the work, and approved it for publication.

## Conflict of Interest

The authors declare that the research was conducted in the absence of any commercial or financial relationships that could be construed as a potential conflict of interest.
